# *In Vitro* Analysis of Breast Cancer Cell Line Tumourspheres and Primary Human Breast Epithelia Mammospheres Demonstrates Inter- and Intrasphere Heterogeneity

**DOI:** 10.1371/journal.pone.0064388

**Published:** 2013-06-04

**Authors:** Chanel E. Smart, Brian J. Morrison, Jodi M. Saunus, Ana Cristina Vargas, Patricia Keith, Lynne Reid, Leesa Wockner, Marjan Askarian Amiri, Debina Sarkar, Peter T. Simpson, Catherine Clarke, Chris W. Schmidt, Brent A. Reynolds, Sunil R. Lakhani, J. Alejandro Lopez

**Affiliations:** 1 UQ Centre for Clinical Research (UQCCR), The University of Queensland, Brisbane, Queensland, Australia; 2 The Queensland Institute of Medical Research (QIMR), Brisbane, Queensland, Australia; 3 School of Biomolecular and Physical Sciences, Griffith University, Brisbane, Queensland, Australia; 4 Dept. Neurosurgery, McKnight Brain Institute, University of Florida, Gainesville, Florida, United States of America; 5 Department of Anatomical Pathology, Pathology Queensland: Royal Brisbane & Women’s Hospital, Brisbane, Queensland, Australia; 6 School of Medicine, The University of Queensland, Brisbane, Queensland, Australia; University of California, San Diego, United States of America

## Abstract

Mammosphere and breast tumoursphere culture have gained popularity as *in vitro* assays for propagating and analysing normal and cancer stem cells. Whether the spheres derived from different sources or parent cultures themselves are indeed single entities enriched in stem/progenitor cells compared to other culture formats has not been fully determined. We surveyed sphere-forming capacity across 26 breast cell lines, immunophenotyped spheres from six luminal- and basal-like lines by immunohistochemistry and flow cytometry and compared clonogenicity between sphere, adherent and matrigel culture formats using *in vitro* functional assays. Analyses revealed morphological and molecular intra- and inter-sphere heterogeneity, consistent with adherent parental cell line phenotypes. Flow cytometry showed sphere culture does not universally enrich for markers previously associated with stem cell phenotypes, although we found some cell-line specific changes between sphere and adherent formats. Sphere-forming efficiency was significantly lower than adherent or matrigel clonogenicity and constant over serial passage. Surprisingly, self-renewal capacity of sphere-derived cells was similar/lower than other culture formats. We observed significant correlation between long-term-proliferating-cell symmetric division rates in sphere and adherent cultures, suggesting functional overlap between the compartments sustaining them. Experiments with normal primary human mammary epithelia, including sorted luminal (MUC1^+^) and basal/myoepithelial (CD10^+^) cells revealed distinct luminal-like, basal-like and mesenchymal entities amongst primary mammospheres. Morphological and colony-forming-cell assay data suggested mammosphere culture may enrich for a luminal progenitor phenotype, or induce reversion/relaxation of the basal/mesenchymal *in vitro* selection occurring with adherent culture. Overall, cell line tumourspheres and primary mammospheres are not homogenous entities enriched for stem cells, suggesting a more cautious approach to interpreting data from these assays and careful consideration of its limitations. Sphere culture may represent an alternative 3-dimensional culture system which rather than universally ‘enriching’ for stem cells, has utility as one of a suite of functional assays that provide a read-out of progenitor activity.

## Introduction

Breast cancer research relies heavily on functional assays provided by *in vitro* and *in vivo* models. This includes investigations of the cancer stem cell hypothesis stating that malignant tumours are initiated and maintained by a population of tumor cells that share similar biologic properties to normal adult stem cells. Candidate subpopulations of cancer stem cells (CSCs) can be purified using techniques such as fluorescence-activated cell sorting (FACS), then assayed for stem cell-like properties using *in vitro* clonogenicity, tumoursphere formation and *in vivo* tumourigenicity assays [1], [2], [3], [4], [5],[6]. These are used to demonstrate key attributes of stem cells: self-renewal and multi-lineage potential, which in the case of CSCs infers the ability to recapitulate the heterogeneity of the original tumour [7], [8].

The ability to also expand these subpopulations through other means is seen as an extremely useful tool for breast (cancer) stem cell research. *In vitro* enrichment for normal mammary stem cells in non-adherent, serum-free conditions was first reported by Dontu *et al* [9], varying the method pioneered for neural stem cell cultivation [10]. In these conditions, most cells undergo anoikis whilst rare cells divide and generate spheroid structures - mammospheres. Dontu demonstrated an increased frequency of bi-potent progenitors (defined by the ability to give rise to both luminal and myoepithelial compartments) in spheres compared to the original dissociated tissue, and also that mammosphere immunophenotype was consistent with enrichment for dedifferentiated cells [9].

Sphere formation was then reported in cells from primary breast tumours [3], [11], metastases [12] and established cell lines [3], [6], [12], [13], [14] and spheres were shown to be enriched for CSC phenotype measured by increased tumour take rate in *in vivo* xenograft assays. Early reports on the lack or loss of markers of differentiated epithelium (Cytokeratins CK5, CK18, CK19, C14, MUC1, EpCAM and CD10) in spheres [3], [9], [12] may have promoted the idea of a dedifferentiated state for the entire structure. Ponti originally reported a high frequency of CD44+/CD24− cells within spheres [3]. This phenotype suggested enrichment with CSCs as defined by Al-Hajj [1], and more recently this phenotype has been shown to be increased in spheres versus matched adherent cultures of MCF7 cells [15]. Additionally, Cariati indicated that sphere-forming efficiency, and therefore CSC frequency, increases with serial passage in cell lines [6]. Taken together, these lines of evidence suggested that propagation and passage as tumourspheres enriches for breast CSCs, and that the study of whole spheres (e.g. using genome-wide expression platforms [16]), could be exploited to identify new CSC markers and targetable mechanisms underlying stem cell activity.

The search for markers of breast CSCs has intensified over the last decade, with several markers identified in different contexts, but so far this research has not uncovered a single universal marker. This is thought to be due to molecular and cellular heterogeneity and different possible histogenic pathways to breast cancer, reflected in the heterogeneity of breast cancer and their cell line derivatives. The sphere assay itself, has also been used to identify potential breast CSC regulators through characterisation, overexpression, knockdown or antagonist studies. These studies have identified HER2 [4], [17], CD49f [6], PTEN [18], EpCAM [1], [13], ALDH1 (Aldehyde Dehydrogenase 1) [2], [14], AC133 [14], DLL1 and DNER [19] as potential breast CSC regulators. The positive correlations found between increased tumoursphere formation, ALDH-expression and *in vivo* tumourigenicity and interpreted as an enrichment in cancer stem/progenitor cells, as is the case of PTEN knockdown [18] for example, might suggest a strong and simple surrogacy between these functional assays and correlation to stem cell frequency.

Such selected findings, however, have possibly overstated our understanding of the tumoursphere, the biological significance of which is still debatable. These issues have recently been reviewed in historical context, highlighting some of the technical and biological limitations that have accompanied adaptation of the traditional neurosphere assay to model stemness in other tissues [20]. To date, most breast cancer cell line sphere studies have used too few cell lines to reliably interpret data in the context of the heterogeneity of cell line phenotypes [3], [6], [12]. Furthermore, contradictory evidence exists as to the degree of differentiation and heterogeneity within both breast tumourspheres and normal mammospheres [9], [11]. Some studies have shown an inability to serially cultivate normal mammospheres beyond five passages [21], whilst others have demonstrated an inability to maintain a high ratio of CSCs (CD44^+^/CD24^−^) in long-term sphere culture, suggesting limited self-renewal capacity [22]. Spheres are known to express markers of differentiation [9], further suggesting they may not universally ‘enrich’ for stem cells. Other studies are emerging that convincingly demonstrate sphere formation actually reverses CSC phenotype in some cell lines [23]. Furthermore, it is not clear in the current literature whether sphere structures are single entities common to both normal and cancer contexts, or whether daughter cells comprising spheres are any different to daughter cells propagated in other contexts, and if so, are these difference due to their derivation from a stem cell, or simply their 3D architecture in the non-adherent environment?

In this study we aimed to better understand the functional utility and delineate the limitations of sphere assays in the context of breast biology. We investigated sphere-forming capacity in a large heterogeneous panel of breast cancer cell lines and performed detailed characterisation of their morphologies and expression of phenotypic markers (including previously reported markers of differentiated mammary epithelia and CSCs). Data also includes characterisation of the morphological and phenotypic differences within primary mammospheres in a basal−/luminal-like dichotomy.

## Materials and Methods

### Propagation and Culture of Tumourspheres from Established Breast Cancer Cell Lines

The adherent growth conditions of the cell lines used in this study are detailed in Table S1. All cell lines were obtained from the ATTC with the exception of SVCT and Hs578T from ECACC, whilst KPL-1 [24] and PMC-42ET [25] were kindly donated Professor Rik Thompson (St. Vincent’s Hospital, Melbourne). All cell lines were authenticated by STR profiling.

To generate tumourspheres, cells were trypsinized from adherent starter cultures with TrypLE (Gibco), quenched in normal growth media, washed three times in large volumes of calcium-magnesium-free PBS to remove as much serum as possible, then passed through a 40 µm cell strainer (BD Falcon). Cell concentrations were determined using the Countess™ automated cell counter (Invitrogen) then seeded in sphere-promoting culture [26] at densities of 1–5×10^4^ cells/mL in low-adherent 6-well plates, or 5–10×10^4^ cells/mL in low adherent T-75 flasks (Nunc Thermofisher Scientific). NSA media consists of DMEM/F12 (Invitrogen) containing recombinant human epidermal growth factor (EGF; Sigma; 20 ng/mL), recombinant human basic fibroblast growth factor (bFGF; R&D Systems; 10 ng/mL), heparin (Sigma; 4 µg/mL), human or mouse proliferation supplement (NeuroCult®; Stem Cell Technologies; 10%), bovine serum albumin (BSA; Sigma; 0.15%), and penicillin G-streptomycin solution (Gibco; 1%). Cells were grown at 37°C in a humidified atmosphere containing 5% CO_2_. The overlay method was used for matrigel culture in 96 well plates, whereby cells in suspension were seeded in their appropriate normal growth media containing 4% growth factor reduced matrigel (BD) over 50 µL of 100% matrigel previously set after 30 minutes incubation at 37°C. For clonogenicity experiments, parallel adherent, matrigel and sphere cultures were plated at limiting dilution appropriate for each format (40, 4000 and 800 cells/cm^2^ respectively) and resulting clones/structures/spheres were fixed and counted after 7 days. When secondary clonogenicity was performed, spheres were harvested and dissociated as described below, whilst matrigel structures were recovered from the matrix with Dispase (BD) prior to single cell dissociation. Cells were then counted and reseeded in limiting dilution adherent culture as above.

### Primary Human Mammary Epithelial Cell Dissociation and Culture

Normal human breast tissue was obtained from consenting women undergoing reduction mammoplasty surgery. Patients gave written consent for the use of their tissue in research and this was approved by the appropriate local Human Research Ethics Committees: the University of Queensland and the Wesley Uniting Hospital. The tissue was dissociated as previously described [27] and the epithelial-rich component was cultured for 7 d in E93 media: F12 media (Gibco), foetal calf serum (FCS; Gibco; 5%), antibiotic/antimycotic (Gibco; 1x), EGF (10 ng/mL), Insulin (Sigma; 5 µg/mL), Hydrocortisone (Sigma; 1 ug/mL) and cholera toxin (Sigma; 100 ng/mL). Cells were then separated by FACS (see below). Sorted cells were seeded in 6-well plates in triplicate at (5×10^4^/well) in either adherent (E93 media) or sphere-promoting (Mammocult; Stem Cell Technologies) conditions on Poly-HEMA (Sigma)-coated plates. After 10 d in culture, spheres were harvested for immunohistochemical (IHC) analysis or dissociated for clonogenicity assays. Briefly, spheres were collected in 40 µm sieves and then dissociated alongside parallel adherent cultures as above with versene and TrypLE. Resulting single cell suspensions were counted and seeded at 4.5×10^3^ cells/60 mm plate along with 2×10^5^ irradiated NIH 3T3 feeder cells according to the method previously described [28]. After 7 d, cultures were fixed in methanol, stained with Geimsa and colony morphologies assessed by light microscopy.

### Sphere-forming Efficiency Assays

Sphere forming capacity (SFC) was assessed at least twice for each of the cell lines listed in Table S1. Cells were seeded in triplicate in low-adhesion 6-well tissue culture plates (2–5×10^5^ cells/well) in sphere-promoting conditions. The presence of spheres (3D multicellular structures greater than 40 µm in diameter) was assessed by light microscopy after 7 d, and scored based on the number of spheres relative to the number of parent cells seeded: ‘−’ = no spheres observed, ‘+’ = <0.01% and ‘++’ >0.01%. We defined the presence of spheres in a manner similar to described in Maguer-Satta et.al., 2011 [29] as clearly three-dimensional spherical-like groups of cells growing as dense, floating and compact clusters, greater than 40 µm in diameter and clearly distinguishable from loose aggregates of cells.

The sphere-forming efficiency (SFE) of six cells lines at passage one was assessed by culturing set numbers of single cells in 50 µL of NSA media in the wells of a 384 well optical bottom plate (Nunc) (5×10^3^, 3×10^3^, 1×10^3^, 5×10^2^, 3×10^2^ or 1×10^2^/well). After 6-7 d in culture, the number of spheres (>40 µm diameter to discriminate from cell clumps, as above) was counted and used to assess the proportion of spheres formed relative to the number of single cells seeded initially.

### Sphere Size Determination and Photomicrographs

**Figure 1:**
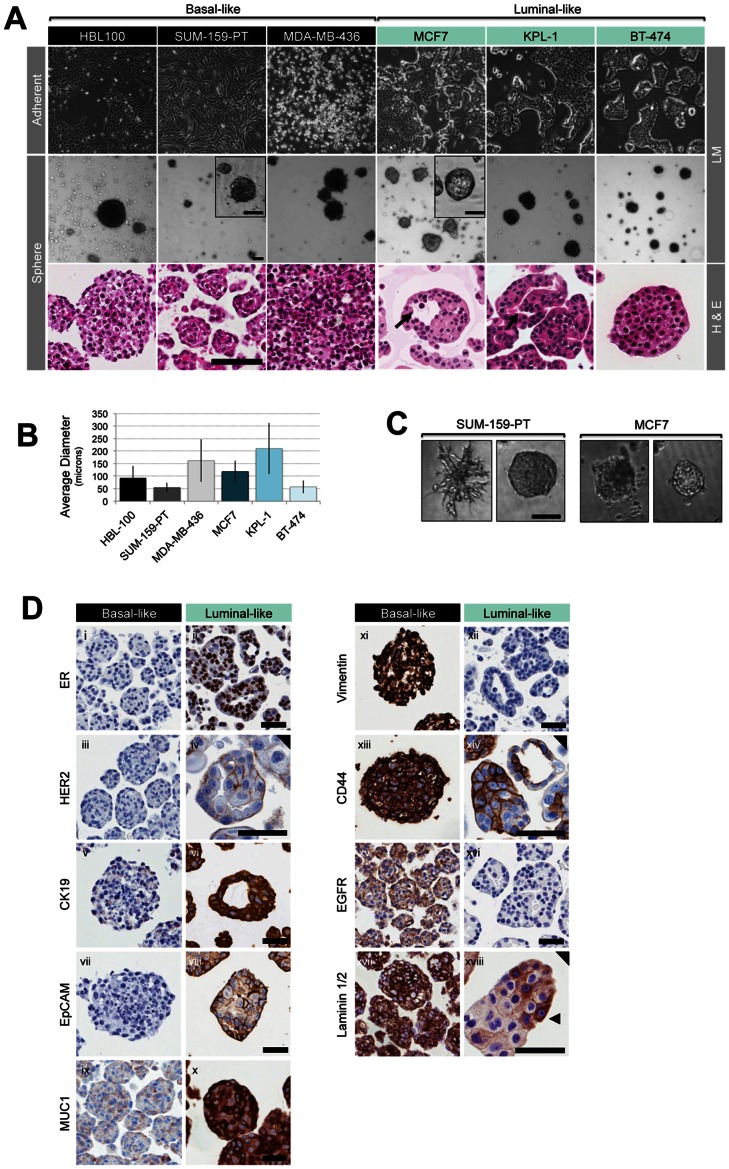
Morphologic and immunohistochemical analysis of tumourspheres cultured from a panel of basal-like/claudin-low and luminal-like breast cancer cell lines. (A) Light microscope images of cell lines grown in adherent and sphere-promoting suspension conditions 7 d after seeding (images taken at 40× magnification, insets 100x magnification). H&E analysis of sections of formalin-fixed, paraffin embedded spheres are also shown. Arrows indicate wide lumina within MCF7 spheres and cleft-like lumina within KPL-1 spheres. Scale bars 100 µm. (B) Average sphere diameter measured by digital image measurement of the long axis of spheres. Data represent median diameter of spheres ± SD measured in 10 random fields, representative of two biological replicates. (C) Light microscope images of structures formed by MCF7 and SUM-159-PT cells grown in matrigel. Images taken at 100x magnification, scale bar 100 µm. (D) Representative images from immunohistochemical analysis of the indicated antigens on FFPE preparations of spheres from basal-like (i, iii, ix, xv, xvii: SUM-159-PT; v, vii, xi, xiii: HBL100) and luminal-like cell lines (ii, vi, xii, xiv: MCF7; iv, viii, xvi, xviii: KPL-1; x: BT-474). Complete data set available in Figure S1. Images taken at 200x magnification (black triangle, 400x). Scale bar 100 µm. Black arrows indicate intermittent laminin1/2.

For sphere sizing, cells were seeded at 1×10^6^ in low-adherent T75 culture flasks. 10 random fields/flask were digitally imaged 7 d after seeding, using a digital camera on a CK40 light microscope (Olympus Corporation). Sphere diameters were measured on the long axis using Photoshop CS3 (Adobe). The average size of spheres in the 10 fields or from n = 100 spheres (whichever was greater) was calculated. Measurements were taken in two independent assays and representative results are shown in Fig. 1.

### Immunohistochemical Phenotyping of Spheres

After 5 d in culture, spheres were pelleted and fixed in 10% neutral buffered formalin for 1 h before being processed for paraffin embedding. 4 mm Sections were cut for standard Haematoxylin & Eosin (H&E), Periodic Acid Schiff PAS stains or prepared for IHC using different antibodies and antigen retrieval methods (Table 1). The following antigen retrieval methods were used: heat retrieval in a decloaking chamber (Biocare Medical) with 0.001 M Tris/ethylenediaminetetraacetic acid (EDTA) pH 8.8, at 105°C for 15 min or 0.01 M citric acid buffer pH 6.0, at 125°C for 5 min; 0.1% Chymotrypsin in 0.01 M CaCl_2_+0.05 M Tris buffer, pH 7.8 at 37^o^C for 10 min. Two detection kits were used: Dako EnVision+ (Dakocytomation) and Vectastain® Universal ABC kit (Vector laboratories) according to the manufacturer’s instructions. Dako HercepTest™ kit was used for Her2 staining. Sections were reviewed by two independent observers and described by a qualified pathologist (ACV). At least 2 independent sphere preparations were observed for each antigen.

**Table 1:** IHC methods.

Antigen	Supplier	Clone	Dilution	Antigen Retrieval	Detection Method
CD44	Dako	DF 1485	1∶100	EDTA	Dako
CK5	Covance	AF138	1∶500	Citrate	Mach1
CK14	Novocastra	LL002	1∶40	Citrate	Mach1
CK19	Dako	BA17	1∶40	Citrate	Vector
E-cadherin	Novocastra	36B5	1∶100	Citrate	Dako
EGFR	Invitrogen	31G7	1∶100	Chymotrypsin	Dako
EMA (MUC1)	Dako	Clone E29	1∶50	EDTA	Vector
ER	Dako	1D5	1∶100	EDTA	Dako
ESA (EpCAM)	Novocastra	Clone VU-1D9	1∶30	EDTA	Dako
Her2	Dako				HercepTestTM
Ki67	Dako	Clone MIB-1	1∶400	Citrate	Vector
Laminin 1+2	Abcam		1∶300	Chymotrypsin	
Vimentin	Dako	V9	1∶400	Citrate	Vector

### Determination of Stem Cell Symmetric Division

Ten breast cancer cell lines representing five luminal-like (MCF7, KPL-1, BT-474, SK-BR-3 and T47D), five basal-like (SUM-159-PT, MDA-MB-436, HBL-100 and Hs578T all Basal B/claudin-low and Basal A BT-20) cell lines were cultured in adherent and sphere-promoting conditions. Nine lines were cultured at 2.5×10^5^ cells/T25 culture flask in triplicate and the total number of cells was calculated every 5 d for 2–8 passages. BT-474 cells were cultured at 1×10^6^ cells for sphere-promoting conditions and 8×10^5^ cells for adherent conditions in T75 culture flasks in triplicate and total number of cells was calculated every 7 d for 6–8 passages. The rates of long-term proliferating (LTP) cell symmetric division (K*_ll_*) was calculated based on fold expansion, using methods previously described [30]. Briefly, fold-expansion represents the cell count at the start of culture divided by the cell count at the end of culture. Symmetric division rate of LTP cells represents the natural log of the fold expansion divided by the time in culture (5 or 7 d). The LTP cell symmetric division reported in this manuscript for KPL-1, MCF7 and BT-474 spheres also appears in the manuscript by Deleyrolle *et al.* [30], however a different analysis is performed within this manuscript.

### Flow Cytometry – Staining and Data Acquisition

**Figure 2:**
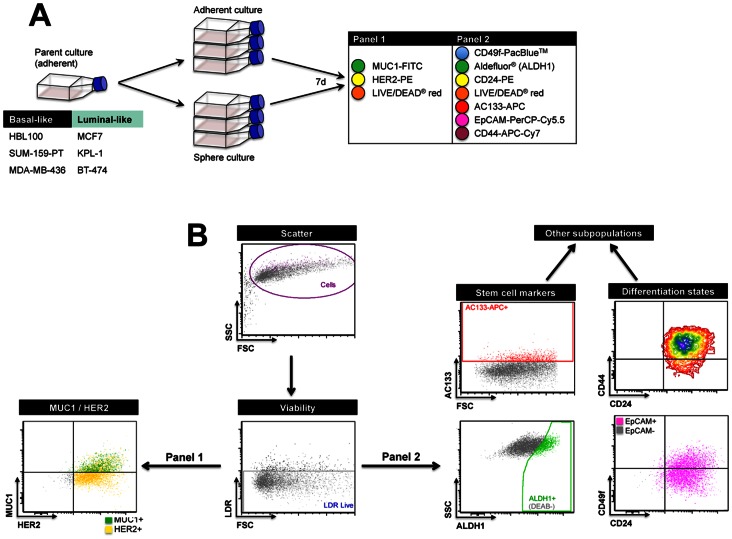
Multiparametric flow cytometry analysis of mammary epithelial and stem cell markers in matched adherent and sphere cultures of breast cancer cell lines. (**A**) **Experiment design.** Matched adherent and sphere cultures of luminal-like and basal-like/claudin-low cell lines were generated in triplicate from subconfluent, adherent parent cultures on d0, harvested and dissociated on d7 into single cell suspensions, then concurrently stained with combinations of fluorescent antibody conjugates. Panel 1: MUC1-FITC, HER2-PE and the LIVE/DEAD® red cell viability stain; Panel 2: CD49f-Pacific Blue, Aldefluor® reagent (for ALDH1 activity), CD24-PE, LIVE/DEAD® red, AC133-APC, EpCAM-PerCP Cy5.5 and CD44-APC-Cy7. (**B**) **Gating strategy for simultaneous detection of cell surface markers.** Acellular particles and dead cells were excluded based on low light scatter and LIVE/DEAD® red positivity (live cells designated ‘LDR live’). For each experiment, gates were placed based on autofluorescence of unstained adherent or sphere control samples to account for differences in the autofluorescence of cells grown in the different formats. For Aldefluor® (ALDH1 activity), gates were placed based on the fluorescence of the DEAB negative control (refer to supp methods). Population frequencies were determined for individual parameters (MUC1+, HER2+, AC133+, EpCAM+, ALDH1+, CD44+, CD24+, CD49f+). For panel 2, combination gating was performed to investigate the frequencies of stem cell populations, differentiation states and other subpopulations (see Fig. 3).

Matched adherent and sphere cultures of luminal and Claudin-low cell lines were generated in triplicate (technical replicates) from subconfluent, adherent parent cultures on d0, harvested and dissociated on d7 into single cell suspensions, then concurrently stained with combinations of fluorescent antibody conjugates for simultaneous detection of cell surface markers (Fig. 2). Panel 1: MUC1-FITC (BD), HER2-PE (BD) and the LIVE/DEAD® red cell viability stain (Invitrogen); Panel 2: CD49f-Pacific Blue (Biolegend), Aldefluor® assay reagent (StemCell Technologies), CD24-PE (BD), LIVE/DEAD® red, AC133-APC (Milltenyi), EpCAM-PerCP Cy5.5 (BD) and CD44-APC-Cy7 (Biolegend). Staining with the Aldefluor® assay for detection of ALDH1 activity was done prior to the addition of fluorescent antibodies according to the maufacturer’s instructions. Each Aldefluor-containing sample was prepared in duplicate, and the second negative control sample was immediately quenched in the ALDH1 inhibitor Diethylaminobenzaldehyde (DEAB) as per the manufacturer’s instructions.

**Figure 3:**
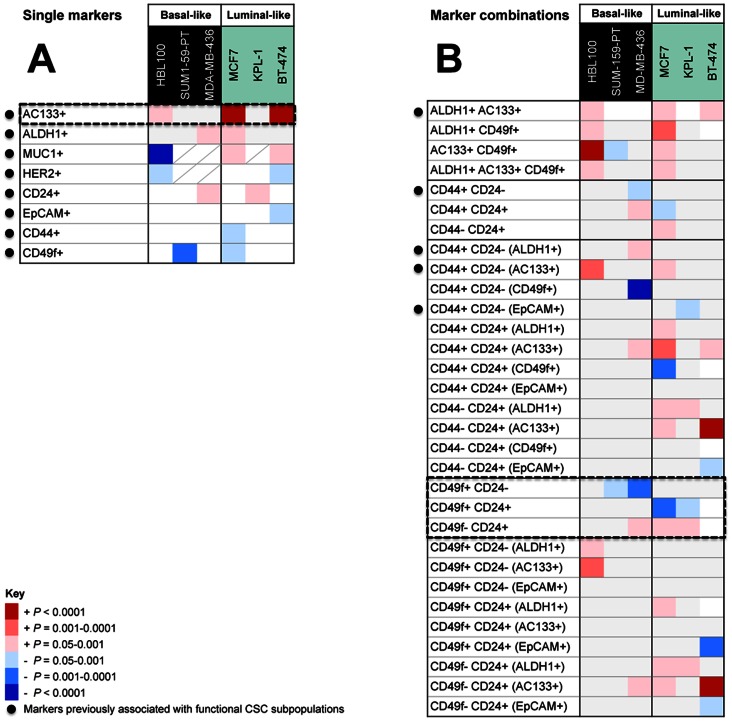
Differences in the frequencies of functional mammary epithelial subpopulations in parallel sphere and adherent cultures of breast cancer cell lines assessed by multiparametric flow cytometry analysis. Adherent and sphere cultures were dissociated, stained with fluorescent antibody conjugates and analysed as described in Fig. 2. Heat maps show changes in subpopulation frequencies within the live cell population. Red shades = higher frequency in spheres compared to matched adherent cultures; blue shades = lower frequency in spheres; empty = not consistent and/or not statistically significant across biological replicates; diagonal line = not determined. Statistical significance was determined using two-way ANOVA and 2-tailed, paired t-tests. Results are depicted only where the trend was directionally consistent and statistically significant over biological replicates (refer to methods). Significance levels are colour-coded: light pink/blue: *P* = 0.05–0.001; mid-pink/blue: *P* = 0.001–0.0001; dark red/blue: *P*<0.0001). Dotted boxes indicate changes of interest: significant enrichment with AC133+ cells in 3/6 cell lines (A), and frequent shifts in CD49f^+^/CD24^+^ phenotypes with sphere culture (B).

Raw fluorescence data was collected on a FACSAria I flow cytometer (Becton Dickinson) using FACSDiva acquisition software (v6.1.3; BD). Particles and dead cells were excluded based on low light scatter and LIVE/DEAD® red positivity. 1×10^4^ Events that fell within the live cell gate were collected for each sample. Manual fluorescence compensation was performed on each occasion, then retrospectively checked and modified if necessary using FCS Express analysis software (v4.0; DeNovo Software). For each experiment, gates were placed based on unstained adherent or sphere control samples to account for variations in the autofluorescence of cells grown in the different formats. For Aldefluor®-stained samples, gates were placed based on the fluorescence of parallel samples stained with the ALDH1 inhibitor, DEAB. Population frequencies were determined for individual parameters. For panel 2, combination gating was performed to investigate the frequencies of stem cell populations, differentiation states and other subpopulations (Figure 3/4). Samples stained with the relevant isotype controls were checked to ensure best gate placement, and ‘fluorescence minus one’ (FMO) controls were checked to ensure the fluorescent panels chosen could be accurately compensated.

**Figure 4:**
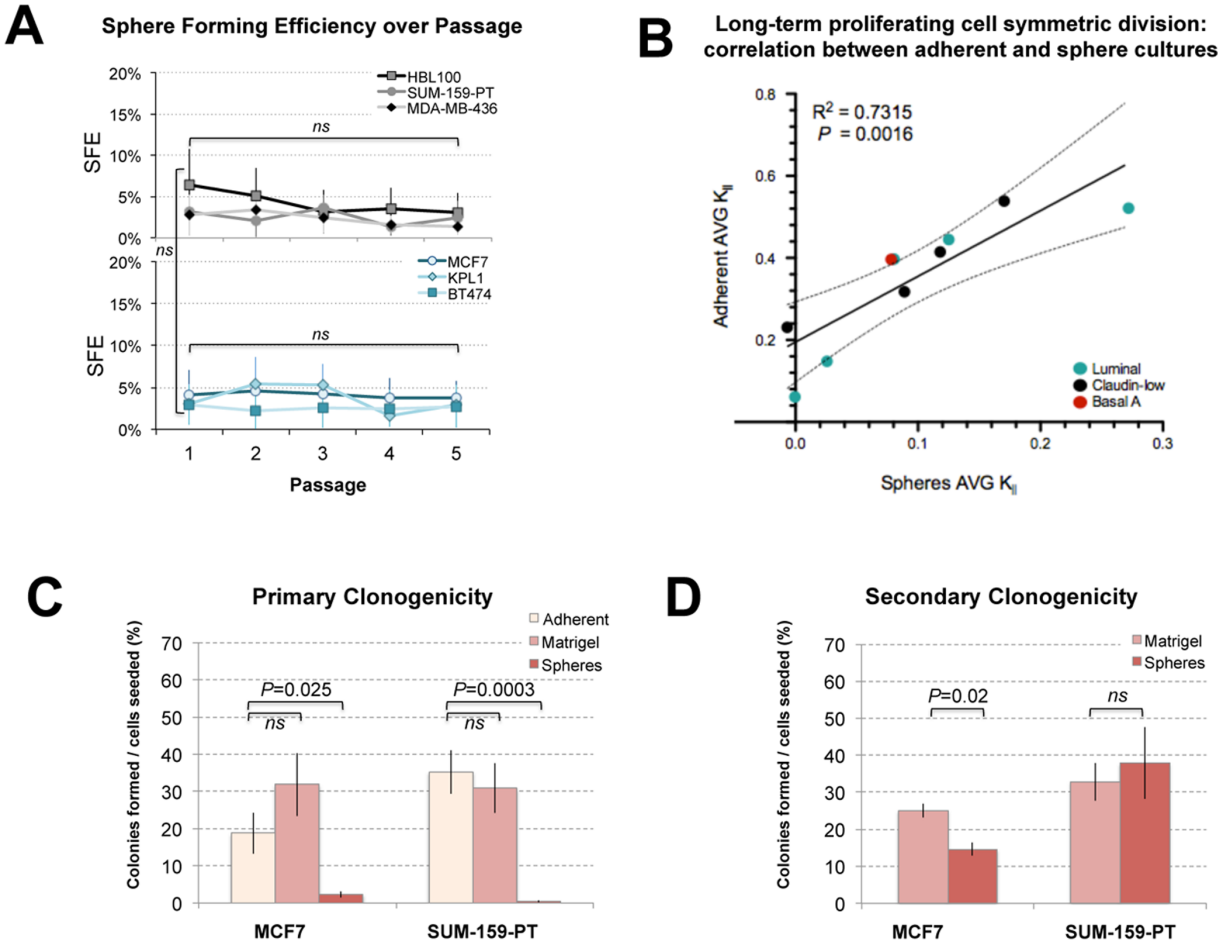
*In vitro* functional analysis of progenitor cell content in breast cancer cell line tumourspheres. **(A) Sphere forming efficiency (SFE) of cell lines through serial sphere passage.**–14 replicates per dilution, 4 dilutions/cell line/passage), expressed as a percentage of the original number of cells seeded, then averaged across the dilutions. Bars represent the mean SFE ± SD from 2–3 independent experiments. Two-tailed, unpaired t-tests demonstrated no significant difference in SFE between passage 1 and 5 for any cell line (*ns*). (**B**) **Correlation between stem cell symmetric division rates of matched adherent and sphere cultures.** Ten breast cancer cell lines (MCF7, KPL-1, BT-474, SKBR-3, T47D, SUM-159-PT, MDA-MB-436, HBL100, Hs578T and BT-20) were seeded in triplicate at equal densities in adherent or sphere-promoting conditions, and the total number of cells was calculated every 5–7 d for 2–8 passages. Mean fold expansion and the rates of long-term proliferating cell symmetric division (K*_ll_*) were calculated for each culture [30] and plotted for correlation analysis (solid line, linear correlation; dotted lines 95% confidence interval). The relationship between adherent and sphere K*_II_* rates was statistically significant (linear correlation analysis (post-test for linear trend); *P* = 0.0016). (**C**) **Primary clonogenicity of MCF7 and SUM-159-PT cells in different growth formats.** Clonogenicity in adherent growth conditions, matrigel overlay and sphere-promoting conditions was calculated as the number of colonies (adherent or non-adherent) that grew after 7 d, as a percentage of cells seeded. Data shown are means ± SE, representative of at least three separate experiments performed in triplicate. (**D**) **Secondary clonogenicity of MCF7 and SUM-159-PT cells derived from spheres and matrigel structures.** Clonogenicity was calculated after dissociating and reseeding cells derived from spheres and matrigel structures into adherent conditions at limiting dilution. Data are means ± SE, representative of one of three separate experiments performed in triplicate. *P* values in C and D were generated using two-tailed, unpaired t-tests.

### Flow Cytometry – Statistical Analysis

Further data analysis was performed in the R statistical environment. First, outliers were removed if they represented obvious deviations from the overall pattern of the data. Outliers were defined as datapoints 1.5x the interquartile range above the 3^rd^ quartile or below the 1^st^ quartile of the biological and technical replicates combined, and comprised ∼1.2% of the dataset. Differences between control (adherent) and test (sphere) means were determined for each biological replicate (minimum 2, usually 3, performed in triplicate).

A two-stage statistical analysis was then applied to the data to determine which parameters were significantly and directionally consistently altered with sphere culture. 1) A two-way ANOVA test was applied: a) to exclude parameters for which there was a significant treatment experiment interaction (*P*<0.01, indicating a lack of reproducibility between biological replicates); and b) to calculate the statistical significance of consistent changes (‘treatment effects’) considering (data from all experiments where *P*<0.05). 2) Whilst the ANOVA procedure is useful for filtering irreproducible data, it also excluded datapoints where the differences between treatments and controls for a set of biological replicates were consistent in direction (i.e. all increases or decreases), but not in magnitude. Given we were interested in all directionally consistent changes regardless of magnitude, datapoints with significant treatment experiment interactions were cross-referenced against two-tailed t-tests for independent biological replicates, and included as significant if at least 2 out of 2 or 3 biological replicates were consistently and significantly altered with sphere culture (*P*<0.05). Data were then finally reviewed, and any datapoints with borderline significance that were based on population frequencies <0.25% were removed.

### Primary Breast Epithelial Cell Sorting

Human mammary epithelial cells (hMECs) were harvested by pre-treatment with versene (Gibco) and TrypLE before washing and passing through a 40 µm filter. Cells were then adjusted to 1×10^6^/mL, and incubated with SYTOX Blue (Molecular Probes; 1∶1000), CD10-PE-Cy5, MUC1-FITC, CD140b-PE, CD31-PE (all from BD Biosciences at the pre-optimised dilution of 1∶100) for 10 min on ice. Live, CD140b-and CD31-negative cells were sorted for MUC1 and CD10 positivity on a FACS ARIA cell sorter, collected in Hanks buffered salt solution containing 2% FCS, then seeded at 1–5×10^4^ cells/mL onto poly-HEMA-coated plates.

## Results

### No Correlation between Tumoursphere-forming Capacity and Breast Cancer Cell Line Subtype, Tumourigenicity or Growth in Matrigel

In order to better understand the biological significance of sphere formation and determine which features might predict for this ability, we tested the abilities of 24 breast cancer and 3 normal breast epithelial cell lines (HBL100, MCF10A, SVCT) to form spheres by seeding at low dilution into non-adherent conditions in serum-free media containing EGF and FGF. Sphere formation was observed in 13/26 cell lines. Comparison to molecular phenotypic data from other studies [31], [32], [33], [34] revealed no clear correlation with sphere formation (Table S1). Non-sphere-forming and sphere-forming cells lines fell in similar proportions amongst luminal-like and basal-like categories [32], [34]. Collation of data on cell line *in vivo* tumourigenicity using various mouse xenograft models [31], [35] also failed to stratify cell lines into sphere-forming and non-sphere forming categories, as did morphological phenotypes in matrigel [36] indicating that the relationships between CSC frequency/activity, molecular phenotype, tumourigenicity and sphere-forming efficiency are complex.

### Distinct Sphere Morphologies and Immunophenotypes in Basal- and Luminal-like Breast Cancer Cell Lines

Unable to predict sphere-forming ability from the aforementioned properties of adherent cell lines, we decided to more closely examine tumourspheres derived from a smaller panel of breast cancer cell lines, with a view to exploring the idea that tumourspheres are enriched with CSC phenotypes and/or function. In order to assess sphere formation and characteristics from parental cell-lines of the different major phenotypes, we selected 3 luminal-like and 3 basal-like cells lines, the latter of which are incidentally further subclassified as Basal B [34], [37] or claudin-low [38], previously reported to represent a more mesenchymal phenotype that may be enriched with CSC phenotypes. SUM-159-PT and MDA-MB-346 are both frequently assayed basal-like cell lines harbouring *RB1* and *HRAS* mutations respectively [32]. HBL100 was included as an additional basal-like cell-line, clustering on a molecular level with the BasalB [34] or Claudin-low phenotype [38] cell-lines. It is reportedly derived from normal breast tissue however, controversially harbouring a Y chromosome [39] which has cast doubt on the cell-line’s true origin. We nonetheless, decided to include this interesting cell line with a view to discerning which sphere features might be phenotype or tissue specific. Amongst the luminal-like cell lines, MCF7 and BT-474 are amongst the most frequently used luminal-like cell lines, both harbouring *PIK3A* muations. MCF7 cells exhibit several features of luminal-like breast cancer including retention of ER protein, whilst BT-474 harbour an *ERBB2* amplification [32], [34]. Sphere size was variable both within and between luminal- and basal-like lines, indicating heterogeneous proliferation rates (Fig. 1A/B). Examination of H&E-stained sections revealed several breast tumoursphere architecture categories (Fig. 1A), somewhat reminiscent of 3D structures formed in matrigel [36], [40] (Fig. 1C). HBL100 and SUM-159-PT spheres formed solid structures with irregular edges, comprising cohesive but loosely packed cells. MDA-MB-436 spheres disintegrated upon histological preparation, suggesting unstable or weak inter-cellular adhesion. In contrast to basal-like cell lines, MCF7, KPL-1 and BT-474 spheres comprised tightly packed, cohesive cells within a well-defined border. Whilst BT-474 spheres were spherical solid masses, a proportion of KPL-1 and MCF7 spheres were hollow. KPL-1 structures were consistently of irregular shape and contained inner cleft-like lumina reminiscent of papillary hyperplasia (Fig. 1A). MCF7 spheres exhibited both solid symmetrical and asymmetrical structures with lumina. Mitoses and apoptotic cells were prominent in spheres from all six lines (data not shown).

To investigate molecular heterogeneity and differentiation states between and within the cell line spheres, we performed immunohistochemical (IHC) analysis. Spheres from basal- and luminal-like lines exhibited distinct basal/mesenchymal and luminal IHC profiles respectively (Fig. 1D, Fig. S1). Basal-like spheres were homogeneously negative for CK19, CK14, CK5 (Fig. S2), oestrogen receptor (ER), EpCAM, MUC1 and E-cadherin, and positive for basal markers CD44 and EGFR, and the mesenchymal marker vimentin [32]. Overall, luminal-like spheres demonstrated fidelity to the differentiated luminal phenotype (ER^+^, CK19^+^, E-cadherin^+^, EpCAM^+^, MUC1^+^, vimentin^-^ and EGFR^-^), although unlike basal-like spheres, heterogeneity was observed both between and within luminal-like spheres for ER, HER2, EpCAM, CD44 and MUC1. CD44 expression was concentrated in patches (Fig. 1Dxiv). Similarly, MUC1 showed mostly diffuse cytoplasmic staining, although prominent ‘apical’ or membranous staining was sometimes observed for KPL-1 and MCF7 (Fig. S1). Overall, sphere morphologies and phenotypes reflected the adherent parent lines and did not exhibit gross molecular dedifferentiation as suggested by Ponti [3], although luminal-like spheres exhibited some molecular heterogeneity. Both basal- and luminal-like spheres were capable of secreting their own Laminin extracellular matrix (Fig. 1Dxvii/xviii, Fig. S1k).

### Breast Cancer Cell Line Spheres are not Universally Enriched for Markers of Stem Cell Activity

Subsequent to the discovery that the combination of two markers, CD44 and CD24, can define and enrich for an important stem cell population in breast tumours by flow cytometry [1], others demonstrated that the addition of a third marker to this combination could further purify cells with different functional characteristics, namely EpCAM^+^/CD44^+^/CD24^−^ [13] and Aldefluor^bri^/CD44^+^/CD24^−^ [41]. HER2 [4], [17], CD49f [6], AC133 [14] have also been shown to define or influence the activity of breast cancer stem cell populations and MUC1, in addition to delineating the mature luminal subpopulation of normal cells, has been shown to be overexpressed in a majority of breast cancers [42]. We therefore designed a flow cytometry protocol to simultaneously detect six markers previously shown be important in delineating functional mammary epithelial cell subpopulations (Fig. 2A), enabling detection of a large number of possible marker combinations and subpopulations. We hypothesised that if spheres were enriched for CSC activity or any particular progenitor subpopulation, previously reported CSC markers, or novel marker combination phenotypes, would be more frequent within sphere preparations, which if rare could be difficult to quantitate using standard IHC analyses (Fig. 1D). To assess whether molecularly-defined subpopulations are enriched with sphere culture, we applied two fluorescent antibody conjugate panels (comprising MUC1, HER2, CD49f, CD24, CD44, AC133, EpCAM and Aldefluor®, an enzyme assay that gives a read-out of ALDH1 activity) to screen for any changes in subpopulation frequencies between parallel, 7-day sphere and adherent cell line cultures (Fig. 2). The data are presented as differences in subpopulation frequencies that were reproducible between biological replicate experiments, and statistically significant (Fig. 3; representative raw data in Figs S3 and S4).

Examination of single marker positivity between sphere and adherent cells revealed no consistent changes amongst the 6 cell lines, or across molecular subtypes (Fig. 3A), although cell line-specific changes were observed, reinforcing the idea that spheres are molecularly heterogeneous. ALDH1 activity was enriched in MDA-MB-436 and MCF7 spheres, while CD49f was reduced in spheres for SUM-159-PT and MCF7 (in contrast to a previous report [6]). AC133 positivity was enriched in 3 of the cell lines tested (HBL100, and highly significantly enriched in MCF7 and BT-474 spheres (*P*<0.0001, Fig. 3A dashed box; Fig. S3B)). The significance of this is unclear, but it is noteworthy that AC133 positivity has been associated with a phenotypic shift from bipotency to a committed luminal progenitor state in normal primary human mammary epithelia [43]. Moreover, others have shown AC133 is associated with SLUG expression in primary breast tumourspheres [44], and SLUG is associated with accumulation of luminal progenitor cells and defective luminal lineage commitment in human breast tumours [45].

Examination of marker combinations also revealed variability, with no consistent changes observed across cell lines or within subtypes. We did not observe consistent increases in the frequency of CD44^+^/CD24^−^ cells (nor with further stratification with EpCAM or ALDH1) that we hypothesised may be more frequent in spheres based on previous reports (Fig. 3B). We did observe frequent changes in CD49/CD24 distribution with sphere culture, with significant loss of CD49f^+^/CD24^−^ or CD49f^+^/CD24^+^ cells in 4/6 lines tested (SUM-159-PT, MDA-MB-436, MCF7 and KPL-1; *P*<0.05, Fig. 3B dashed box; Fig. S4B). Given others have shown that CD49f/CD24 distribution can reflect broader states of differentiation defined by multiple biomarkers as well as morphology [46], these data suggest that culturing cell lines as spheres may alter their differentiation programs. Of technical importance, we often observed higher levels of autofluorescence in spheres compared to adherent which might erroneously inflate sphere expression levels if not considered by running comprehensive controls and in threshold and gate application. In order to confirm our observations in BT-474 and SUM-159-PT (Figs S2C and S3B; CD49f), we independently validated these by switching CD49f to an alternative, unaffected fluorochrome (data not shown).

In addition to these findings, we did observe cell-line specific changes for several marker combinations, including spheres from HBL100 (AC133^+^/CD49f^+^), MDA-MB-436 (CD44^+^/CD24^−/^CD49f^+^), and BT-474 (CD44^−/^CD24^+^/AC133^+^ and CD49f^−/^CD24^+^/AC133^+^), which were not only reproducibly observed between biological replicates, but highly statistically significant (*P*<0.0001) and occurred independently of changes in the respective single markers (Fig. 3A). Overall our observations indicate, with the markers we have examined, that there are no consistent global changes in breast cancer cell line spheres including putative cancer stem cell combinations, but that each cell line may undergo its own individual increases or decreases in marker expression when cultured in this format.

### *In vitro* Functional Analysis of Breast Cancer Cell Line Tumoursphere Progenitor Cell Content Compared to Adherent Cells and Matrigel Structures

Since morphologic and immunophenotypic analyses suggested that spheres are not consistently enriched with particular immuno-phenotypes, we explored whether they could be enriched for CSC activity using *in vitro* assays of self-renewal and clonogenicity. Serial sphere passage has been reported to increase sphere-forming efficiency (SFE) in MCF7 cells [6]. We reasoned that if this was a general phenomenon in sphere-forming lines, comparison of early with late passage spheres could be used to identify new CSC markers. We determined SFE in the 6 cell line panel over 5 serial passages, dissociating spheres and reseeding single cells after 7 days. SFEs were similar at first passage (3.0–4.4%, 7% for HBL100), and surprisingly, this was unchanged over serial passage in all lines tested (Fig. 4A), indicating there was no enrichment with sphere-initiating cells, and that comparing early and late passage spheres was unlikely to be a useful approach for studying CSC phenotype or function.

In order to determine whether the cell-lines grew at different rates and therefore exhibit different progenitor frequencies and/or self-renewal rates in spheres or in traditional adherent culture, we compared the fold expansion rates of 10 cell lines grown in both formats in parallel. After applying a mathematic model that uses fold expansion over serial passage to calculate the long-term proliferating cell symmetric division rate (K*_ll_*) [30], we observed a significant correlation between matched sphere and adherent cultures (*P* = 0.0016; Fig. 4B). Essentially this shows growth rate is inherent to the cell line and not the culture format. This raises the possibility that the same progenitor populations could sustain sphere and adherent cultures, but that the symmetric division rate in spheres is restricted by physical limitations (lack of growth factors, matrix attachment and/or biophysical size restrictions). This casts further doubt on the notion of spheres containing an enriched population of cells with different growth characteristics.

We then assessed the relative frequencies of sphere- and matrigel structure-initiating cells compared to standard adherent conditions by calculating clonogenicity (% of cells capable of initiating new clonal growth) after seeding cells at clonal density in the different growth conditions (Fig. 4C). We reasoned this would indicate the frequency of colony forming cells in each culture format. Interestingly, MCF7 clonogenicity was highest in matrigel (43%) compared to adherent (24%), whilst SUM-159-PT was similar (37%) in matrigel when compared to adherent (45%). As expected, clonogenicity in sphere-promoting conditions (SFE) was much lower: 4% for MCF7 and 1% for SUM-159-PT. This suggests that only rare cells are capable of generating full clones in these conditions.

Given the published evidence of increased tumourigenicity from spheres compared to adherent cultures, but having failed to observe any enrichment with self-renewing, sphere-forming cells (Fig. 4A), we decided to analyse enrichment for other progenitor or colony-forming cells. We therefore compared the secondary clonogenicity rates of spheres and matrigel structures, dissociating them and reseeding equal numbers of cells at clonal density in adherent conditions. Surprisingly, both spheres and matrigel structures exhibited secondary cloning rates comparable to primary clonogenicity in adherent conditions (Fig. 4D compared to 4C), indicating that spheres are no more enriched with adherent culture-competent progenitor cells than cells maintained in adherent or matrigel cultures. One interpretation of this is that progenitor cells reside in these structures at similar frequencies.

### Multiple Morphologically and Phenotypically Distinct Entities in Normal Primary Mammary Epithelial Mammosphere Preparations and Enrichment in FACS-sorted Sub-populations

**Figure 5:**
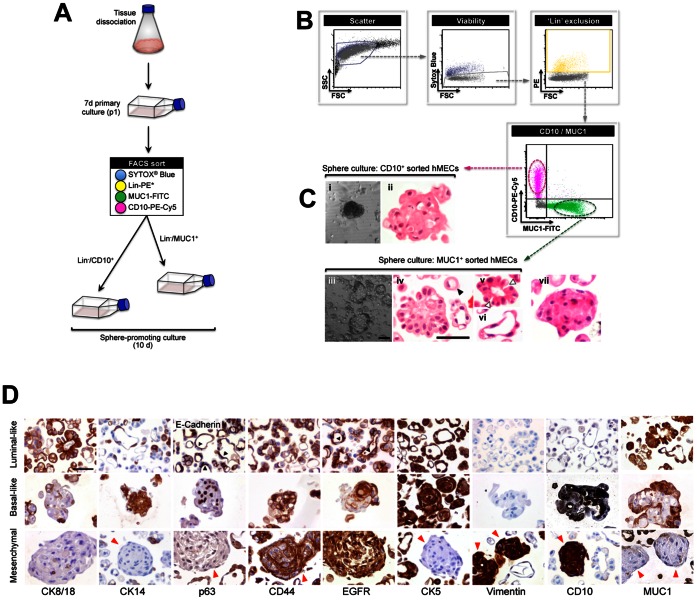
Mammospheres derived from MUC1+ or CD10+ progenitors from reduction mammoplasties comprise hollow and solid structures consistent with luminal- and myoepithelial-like morphologies. (**A**) **Strategy for isolating luminal and myoepithelial progenitor-enriched subpopulations from reduction mammoplasty tissue.** Tissues were physically and enzymatically processed to epithelial-rich, single cell suspensions, then cultured for 7d in mammary epithelial growth medium to generate enough cells for fluorescence-activated cell sorting (FACS) and sphere culture. Primary cultures were stained with fluorescent antibody conjugates (MUC1-FITC, CD10-PE-Cy5), the Sytox® Blue viability stain and ‘Lineage’ cocktail (‘Lin’: CD140b-PE, CD45-PE, CD31-PE; markers of stromal fibroblasts, leukocytes and endothelia respectively). CD10 and MUC1-sorted cells were placed in sphere-promoting culture for 10d before analysis. (**B**) **Gating strategy for enrichment of luminal- and myoepithelial-like progenitor cells.** Acellular particles and dead cells were excluded based on low light scatter and Sytox® blue positivity. Non-epithelial, PE^+^ cells were also excluded, then MUC1^+^ and CD10^+^ cells were collected for sphere culture. (**C**) **Light microscope images (i,iii) and H&E histological sections (ii,iv–vii) of spheres generated from CD10^+^ or MUC1^+^ primary breast epithelial cells.** Open arrows, MUC1+-derived spheres often had limited lateral connections giving a petal-like appearance. Solid arrows, shows single cell with signet ring secretory morphology. (**D**) **Immunophenotypic analysis of MUC1^+^ and CD10^+^ progenitor-derived primary breast mammospheres.** Spheres were generated as described in A/B. Representative images from immunohistochemical analysis of the indicated antigens on FFPE preparations of spheres are shown. Images taken at 200× magnification. Scale bar 100 µm. Black arrows indicate apical membranous staining of E-cadherin and EGFR in luminal-like spheres. Red arrows indicate the mesenchymal-like spheres found rarely amongst the dominant structures formed in both CD10+ and MUC1+ sorted populations.

Having observed heterogeneity in tumourspheres derived from breast cancer cell lines, we decided to investigate whether primary normal human mammary epithelial cell (hMEC) mammospheres also exhibit such diversity or whether they are single entities that can be pooled for molecular analysis. After preliminary observations revealing the presence of solid and hollow mammospheres from fresh dissociations of reduction mammoplasty tissue (Fig. S5), we compared the phenotypes of spheres derived from FACS-sorted hMEC subpopulations enriched with luminal and myoepithelial progenitors, based on expression of MUC1 and CD10 respectively [47] (Fig. 5A–C).

After 10 days in culture, we observed solid spheres derived from CD10^+^ myoepithelial-enriched cultures (Fig. 5Ci) showing distinct squamous metaplasia (Fig. 5Cii) and prominent hollow spheres in MUC1^+^ luminal-enriched cultures (Fig. 5Ciii), confirming that the primary mammosphere is not a single entity. Indeed, histological analysis revealed multiple distinct structures (Fig. 5C). MUC1^+^-derived spheres were either solid (Fig. 5Civ), cuboidal/columnar forming hollow structures with limited lateral connections (giving a petal-like appearance; Fig. 5Cv), spindle shaped forming a flat-like hollow structure (Fig. 5Cvi) or single cells with a secretory signet ring appearance (Fig. 5Civ arrow). IHC phenotyping indicated a luminal-like phenotype for these cells, however the unexpectedly high frequency of expression of EGFR, CD44, CK5 (Fig. 5D) and c-kit (not shown) in these structures was somewhat unexpected and suggested a more luminal progenitor-like rather than a mature differentiated luminal phenotype (Fig. 5D). The solid spheres formed by CD10^+^ cells expressed mostly basal markers (Fig. 5D). The presence of minor CK8/18^+^, CK19^+^ and MUC1^+^ populations may indicate bipotentiality in CD10^+^ sphere-forming cells, which would be consistent with observations of sorted cells grown at colony forming density in adherent culture [47]. These results are supported by similar observations that suggest different sphere types in populations of sorted hMECs from fresh dissociations [48], [49]. Interestingly, in two patients we observed a rare solid structure, lacking squamous metaplasia but with a distinctly vimentin-positive mesenchymal immunophenotype (Fig. 5D) which was similar to our findings for basal-like breast cancer cell line spheres.

**Figure 6:**
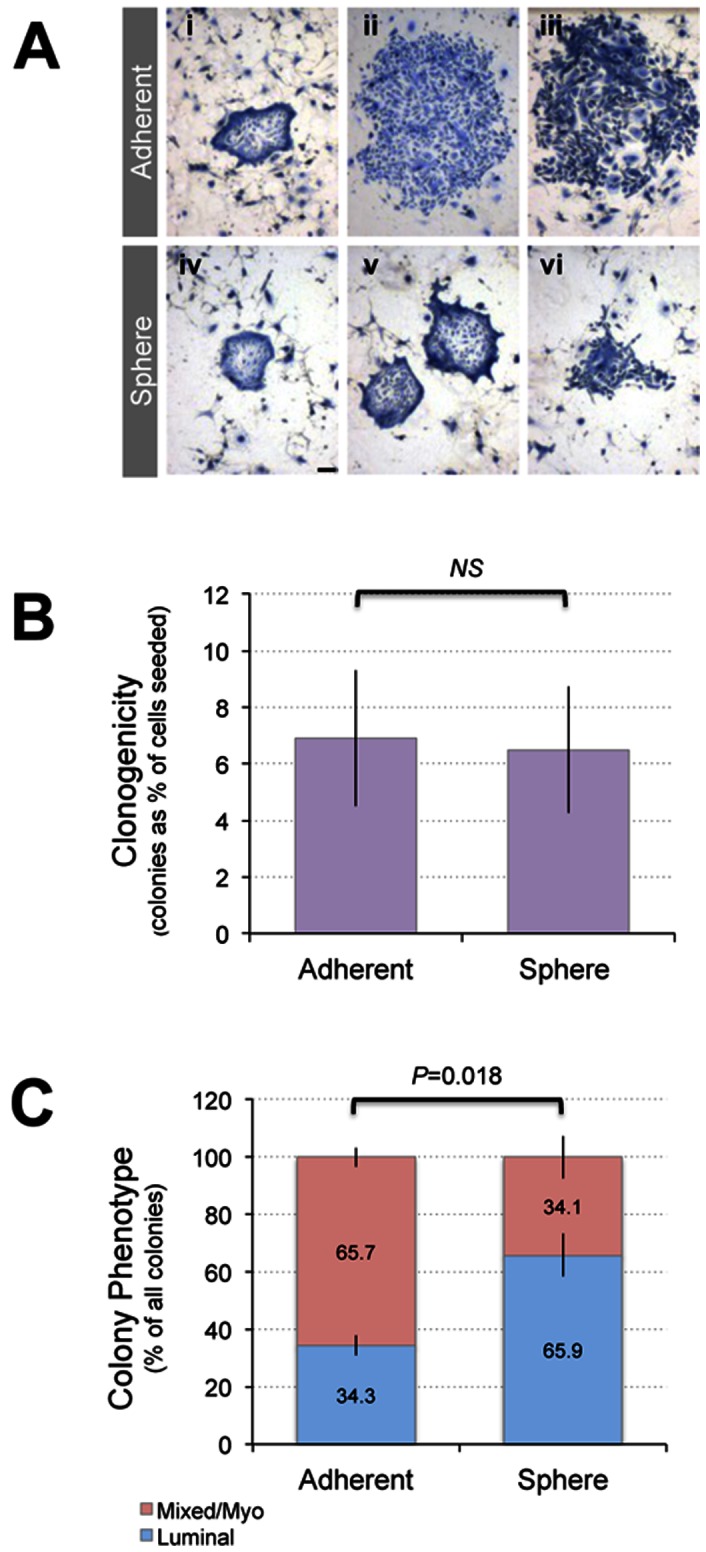
Relative frequencies of luminal and myoepithelial/bipotent human mammary epithelial cells in MUC1^+^-derived mammosphere cultures. MUC1^+^ sphere cultures were prepared as described in Fig. 5A, in parallel with matched MUC1^+^ adherent cultures, then dissociated and used in a colony forming cell (CFC) assay to determine the clonogenicity of luminal and myoepithelial/bipotent progenitor compartments (which give rise to colonies with luminal and mixed/myo morphologies respectively). (A) **Light micrographs of Geimsa-stained colonies** grown from dissociated spheres and parallel adherent cultures. Representative colonies are shown for adherent- (i-iii) and sphere-derived (iv-vi) cells. This includes examples of luminal (i, iv and v), mixed (ii, iii) and myoepithelial (vi) colony morphologies. (B) **Overall clonogenicity** (colonies formed as a percentage of total cells seeded) of spheres and parallel adherent cultures. (C) **Comparison of luminal and myo/bipotent progenitor cell clonogenicity of spheres and parallel adherent cultures.** Data are from four biological replicates (specimens from 4 patients), each performed in triplicate. Statistical tests used were paired, two-tailed students t-tests (*P* values indicated; *ns* = not significant).

If primary mammospheres are enriched for progenitor cells, they might be expected to show increased *in vitro* clonogenicity, and produce a high frequency of colonies with mixed luminal and myoepithelial features, reflecting bipotency [9]. Consistent with this idea, the immuno-phenotype of MUC1^+^-derived hollow spheres (Fig. 5D) suggested enrichment with luminal progenitors. We tested this hypothesis using the colony forming cell (CFC) assay [28], which determines the relative frequencies of major mammary epithelial progenitor types (luminal- and myoepithelial-restricted, and bipotent progenitors) based on the morphologies of colonies that emerge from cells seeded at clonal density (luminal, myoepithelial or mixed respectively; Fig. 6A).

Although it is certainly possible that primary culture itself may affect the cell type frequency and phenotype in the mixed cell population, importantly the parallel sphere and adherent cultures being compared began with the same sorted MUC1+ subpopulation of primary cells. We observed no overall difference in the clonogenicity of MUC1^+^ sorted hMECs grown as spheres or adherent cultures from 4 different patients (Fig. 6B), indicating sphere culture does not enrich for progenitor activity compared to adherent culture. However, we did observe a significant increase in the ratio of luminal to myo/mixed colonies in MUC1^+^-derived sphere compared to adherent cultures (Fig. 6C), suggesting selection of progenitors already locked into a luminal fate or promotion of a luminal lineage differentiation axis. Alternatively, this luminal bias could occur through relaxation or reversion of the adherent *in vitro* selection of basal/mesenchymal states that is thought to occur during the generation of breast cancer and normal breast cell lines [46], providing there is pre-existing luminal progenitor potential in the starting culture.

## Discussion

This study has comprehensively characterised, for the first time, different types (by both morphological and molecular definition) of spheres derived from a panel of 26 breast cancer cell lines and primary normal hMECs. The results conclusively demonstrate that spheres are not homogeneous structures enriched with undifferentiated cells, but rather comprise a range of morphologically distinct entities displaying inter- and intra-sphere molecular heterogeneity, including variable expression of markers of differentiated mammary epithelia. Importantly, we show for the first time that this heterogeneity extends to primary normal mammospheres, and that inter-sphere variability broadly emulates the traditional basal-, luminal- and mesenchymal-like classification system.

Heterogeneity between and within spheres has very important implications, both practical and conceptual. Firstly, sphere preparations contain mixed structures that would likely ambiguate or dilute observations made from pooled, bulk cultures. The data demonstrate that analysis of bulk primary mammosphere cultures in particular, may be no more informative than analyses of a mixture of all primary hMEC types. Interestingly, this dilemma may also apply to more ‘clonally regarded’ cancer cell lines, as Matilainen et al. recently generated two distinct sphere phenotypes from 4T1 cells [23]. Second, the detection of multiple sphere types implies the existence of distinct sphere-initiating cell types. This alerts us to the existence of multiple functional populations within any pool of cells, several of which may be sphere-formers. It also highlights that the ways in which we define functional hMEC subpopulations are limited by our understanding of the biological significance of the various assays originally used for their identification. For example, a particular subpopulation, defined by a defined combination of molecular markers, may show stem cell activity in some assays, and not in others.

The lack of correlation between sphere-forming capacity and known features across our large cell line panel (Table S1), and the variability in expression of key molecular markers observed by comprehensive, multi-parametric flow cytometry profiling (Fig. 3) suggest that we are unlikely to arrive at a universal understanding (or indeed a common set of markers) of the biological significance of sphere formation and its relevance to stem cell phenotype and function. Rather, each cell line and its various subpopulations are unique. These interpretations are supported by work with MCF7 spheres demonstrating a lack of correlation between CD44^+^/CD24^−^ phenotype and sphere formation, tumourigenicity and radiation resistance, with data suggesting separate but sometimes overlapping cell populations [15]. In our multiparametric flow cytometry experiments, we were unfortunately limited in our choice of markers by the commercial availability of fluorescent conjugated antibodies. Further experiments, however, including other markers such as Thy-1 and CD10, recently shown to be important for purifying particular mesenchymal-like stem cell populations in breast cells [48], may be insightful particularly for delineating for mesenchymal and basal-like subpopulations in multiple cell lines.

It is possible that previous reports of positive correlations between stem cell marker frequency, sphere formation, radiation- and chemo-resistance and *in vivo* tumourigenicity may have lead to some overgenerous definitions of CSCs and the suitability of the sphere-assay for functional investigation [3], [4], [6], [18]. Several recent reviews also challenge the biological significance of the assay, and are important reading for any researcher culturing spheres [20], [50]. In a similar vein, Visvader and Lindeman have cautioned, *“…the defining characteristics of these different spheres and their relationship with normal stem cells have been unclear, causing over-interpretation of results… it remains to be determined whether non-adherent spheres selectively enrich for CSCs*” [51].

Our serial passage and primary and secondary clonogenicity assays (Figs 4 and 6) demonstrate that sphere-derived cells are not enriched for colony-forming units, indicating equal, if not reduced, self-renewal in sphere culture compared to adherent and matrigel formats. Moreover, our phenotypic analysis showed spheres expressed multiple markers of differentiation, and were highly similar to their adherent counterparts, suggesting it may be more accurate to regard them as colonies propagated in an alternative, inefficient 3-dimensional culture system, than structures that are enriched with stem cells. Indeed, there are several alternative explanations for data demonstrating increased self-renewal, multilineage potential and tumour initiation from cells derived from spheres [3], [6], [9], which need to be considered.

For example, the behaviour of sphere-derived cells is not routinely compared to an appropriate control (e.g. parallel adherent cultures); and when it is, cell confluency is rarely considered. It is possible that seeding densities and resulting confluency/clone size (or cell per unit volume of media) are very different and not controlled in many published experiments. We have observed that within the usual culture period (5–14 days), the maximum size of a sphere is physically limited, perhaps in response to limited intraspherical diffusion of nutrients and other cell-cell communication processes. Unlike adherent cultures seeded at low density, spheres do not continue to expand into the available space indefinitely (consistent with their proportional, yet lower rate of long-term symmetric cell division; Fig. 4B). This biophysical size limitation may therefore result in significant differences in progenitor frequency within pooled sphere preparations compared to adherent cultures: if each clone is derived from a single initiating stem-like cell that is maintained through passage (Fig. 4A), and sphere clones are smaller, then the progenitor frequency read-out would be higher. Studies comparing tumourigenicity or mammary outgrowth from spheres compared to adherent counterparts where equal total numbers of cells from each format are injected may therefore give false indications of stem cell frequency. Normalising clone size could be one way to overcome this.

A better approach to investigating the relevance of the sphere assay to stem cell function may be to compare spheres with cells from equivalent structures propagated in matrigel, which are similarly restricted in 3D architecture and size. It is well established that the behaviour and phenotypes of cells grown in 3D matrices are different to adherent cultures [36], [52] as better mimics of *in vivo* physiology. *In vitro* cultured multicellular tumour spheroids (MCTS) were first described over 40 years ago [53], and have since been developed by bioengineers as therapy test platforms [54], [55]. They are thought to resemble avascular tumour nodules recapitulating the morphological, functional and mass transport properties of the tissue *in vivo*. In contrast to CSC studies that often focus on measuring stem cell frequency, the primary endpoints of MCTS assays are sphere size and integrity, as indicators of the effect of a test treatment. It is possible that some features of sphere-derived cells currently attributed to stemness may actually reflect size and 3D architecture, which produce very different microenvironmental conditions compared to matched adherent cultures.

Furthermore, our results suggest that in order to truly understand any possible relationship between sphere formation and stemness, measured by tumour initiation or mammary gland recapitulation, the above considerations should preface further *in vivo* studies. Unfortunately outside the scope of the current study, a methodical comparison of these *in vitro* and *in vivo* readouts, with more considered controls (eg. compared to non-sphere culture formats controlled for colony size and 3D architecture) would help disambiguate the biological significance of the sphere assay, or at the very least, better define its limitations.

### Conclusions

In summary, we favour the view that sphere culture could be a system that selects progenitor cells in a phenotypic state permitting growth factor-independence and anoikis resistance, with utility for modelling aspects of breast cancer or mammary differentiation. Extreme caution must be taken, however, in over-interpreting results when the biological significance of the assay is still poorly understood. Our data counters the notions that spheres are single entities and enriched for stem cells. Instead we demonstrate extensive heterogeneity between spheres and the cells that comprise them, implying more complex relationships between sphere formation and other common methods of defining stemness. Studying spheres themselves to understand the biology of normal or CSCs is probably no more informative than studying heterogeneous mammary gland or tumour tissue.

## Supporting Information

Figure S1 **Immunohistochemical analysis of breast cancer cell line spheres.** Immunohistochemical analysis of indicated antigens on FFPE preparations of spheres from three basal- and three luminal-like cell lines. Images were taken at 200x magnification, unless where indicated by black triangle at 400x magnification. Scale bar represents 100 µm. Grey arrowheads indicate areas of HER2 immuno-positivity. Black arrowheads indicate intermittent laminin1/2+ and PAS+ cells in luminal cell spheres. White arrowheads indicate bright PAS staining along the edge of MCF7 and KPL-1 spheres.
(PDF)

Figure S2 **Immunohistochemical analysis of CK5 and CK14 in basal breast cancer cell line spheres.** Immunohistochemical analysis of indicated antigens on FFPE preparations of spheres from three basal-luminal-like cell lines. Images were taken at 200x magnification. Scale bar represents 50 µm.
(PDF)

Figure S3 **Changes in the activity of ALDH1 (A) and expression of EpCAM, CD133 (AC133) (B) and CD49f (C) with sphere culture.** Adherent and sphere cultures were dissociated, stained with fluorescent antibody conjugates and analysed as described in Fig. 2. Representative data are depicted using dot or contour plots. Subpopulation frequencies shown represent the percentage of live cells. Quadrant gates were placed at the threshold of autofluorescence for respective adherent or sphere unstained control samples.
(PDF)

Figure S4 **Changes in cell line differentiation states with sphere culture.** Adherent and sphere cultures were dissociated, stained with fluorescent antibody conjugates and analysed as described in Fig. 2. **(A) Changes in CD44/CD24 phenotypes with sphere culture**. Representative data are depicted using contour plots. Subpopulation frequencies shown represent the percentage of live cells. Quadrant gates were placed at the threshold of autofluorescence for respective adherent or sphere unstained control samples. For KPL-1, the red circle indicates a consistent gain of a CD44^+^/CD24^−^ subpopulation (not statistically significant by statistical analysis of quadrant gates but visually obvious). **(B) Changes in CD49f/CD24/EpCAM distributions with sphere culture**. Where the CD49f/CD24 distribution of EpCAM+ cells differed between adherent and sphere cultures, pie charts indicate the relative proportions of EpCAM+ and EpCAM- cells in each quadrant. 1×10^4^ events displayed on all plots. Red and blue quadrant colouring is transposed from Fig. 3 to indicate subpopulation frequencies that were consistent and statistically significant across biological replicates (red, increased in spheres compared to matched adherent cultures; blue, decreased in spheres).
(PDF)

Figure S5 **Light micrographs of hollow and solid spheres formed from fresh dissociations of normal human breast tissue.** Images were taken at 100x magnification after 10 days in culture.
(PDF)

Table S1 **Breast cancer cell lines: growth media and sphere-forming capacity.** Cell lines were cultured as monolayers in the media indicated. Sphere-forming capacity (SFC) was then determined in triplicate on at least 2 occasions by seeding a standardised number of cells in sphere-promoting conditions (see materials and methods), then counting the number of spheres at 7 d relative to the number of parent cells seeded: ‘−’ = no spheres observed, ‘+’ = <0.01% and ‘++’ >0.01%. SFC was then correlated with adherent growth media, and published data (intrinsic molecular subtypes, tumourigenicity in mouse xenograft assays and 3D *in vitro* morphology in laminin-rich extracellular matrix (lrECM)), however we found no obvious association of any of these parameters with *in vitro* tumoursphere-forming capacity (SFC).
(PDF)
