# Correction: *In Vitro* Analysis of Breast Cancer Cell Line Tumourspheres and Primary Human Breast Epithelia Mammospheres Demonstrates Inter- and Intrasphere Heterogeneity

**DOI:** 10.1371/annotation/1ba8c49a-f6cb-4565-8a36-7d7c429ea670

**Published:** 2013-07-03

**Authors:** Chanel E. Smart, Brian J. Morrison, Jodi M. Saunus, Ana Cristina Vargas, Patricia Keith, Lynne Reid, Leesa Wockner, Marjan Askarian-Amiri, Debina Sarkar, Peter T. Simpson, Catherine Clarke, Chris W. Schmidt, Brent A. Reynolds, Sunil R. Lakhani, J. Alejandro Lopez

The name of the eighth author was given incorrectly. The correct name is: Marjan Askarian-Amiri.

