# Supplementary figures and images for: In Vitro Analysis of Breast Cancer Cell Line Tumourspheres and Primary Human Breast Epithelia Mammospheres Demonstrates Inter- and Intrasphere Heterogeneity
Source: PLoS One. 2013 Jun 4;8(6):e64388. doi: 10.1371/journal.pone.0064388 (PMC3672101; doi:10.1371/journal.pone.0064388)

Figure S1

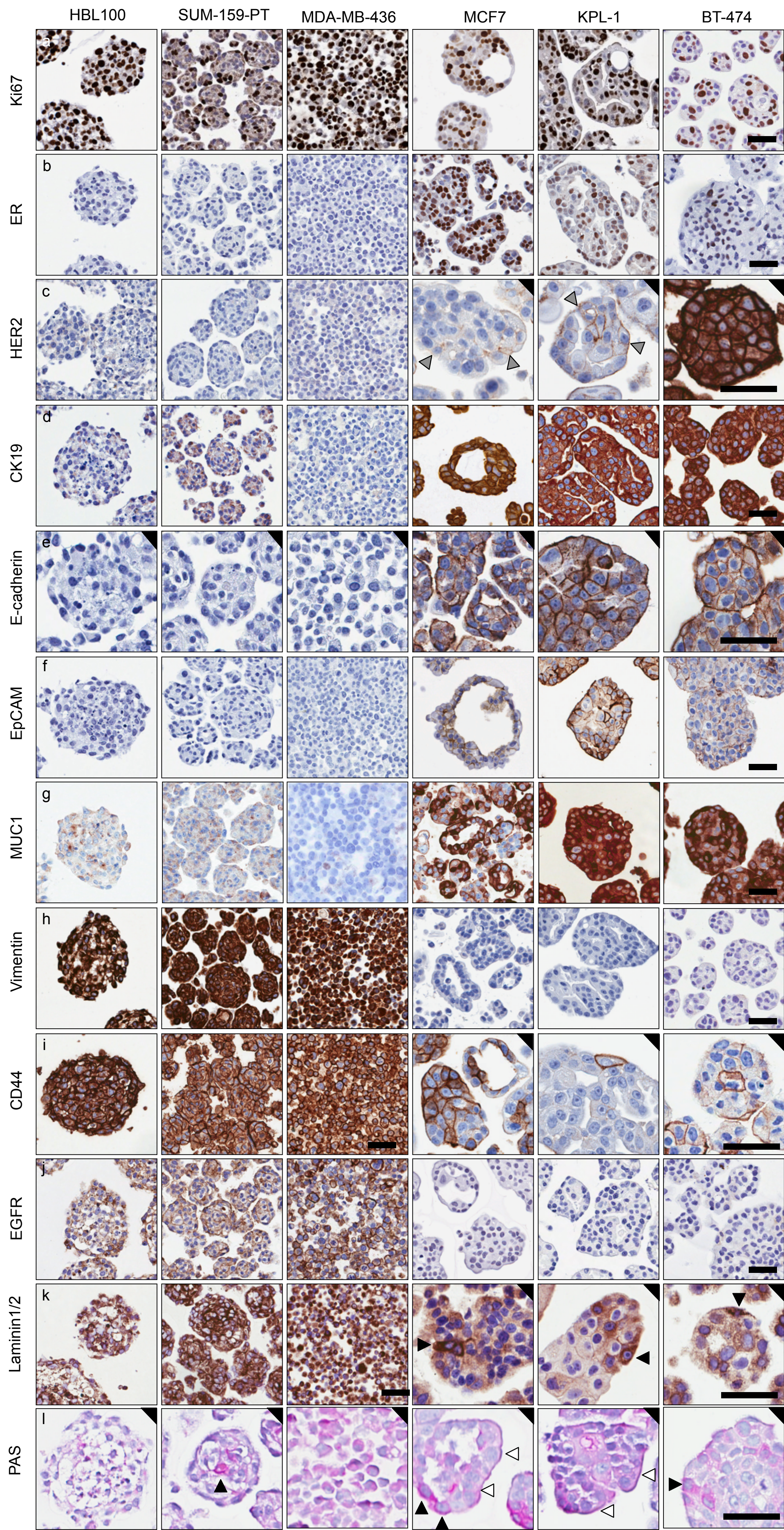

Supplement: Figure S1 — Immunohistochemical analysis of breast cancer cell line spheres. Immunohistochemical analysis of indicated antigens on FFPE preparations of spheres from three basal- and three luminal-like cell lines. Images were taken at 200x magnification, unless where indicated by black triangle at 400x magnification. Scale bar represents 100 µm. Grey arrowheads indicate areas of HER2 immuno-positivity. Black arrowheads indicate intermittent laminin1/2+ and PAS+ cells in luminal cell spheres. White arrowheads indicate bright PAS staining along the edge of MCF7 and KPL-1 spheres. (PDF) [file pone.0064388.s001.pdf]

Figure S2

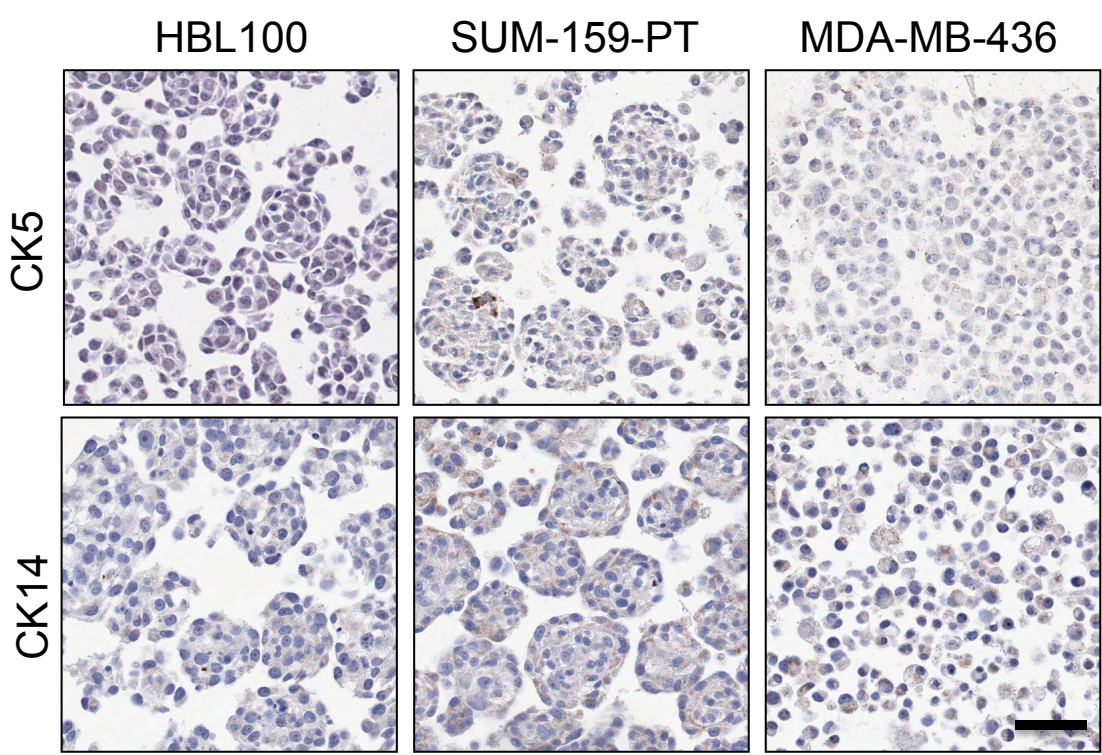

Supplement: Figure S2 — Immunohistochemical analysis of CK5 and CK14 in basal breast cancer cell line spheres. Immunohistochemical analysis of indicated antigens on FFPE preparations of spheres from three basal-luminal-like cell lines. Images were taken at 200x magnification. Scale bar represents 50 µm. (PDF) [file pone.0064388.s002.pdf]

Figure S3

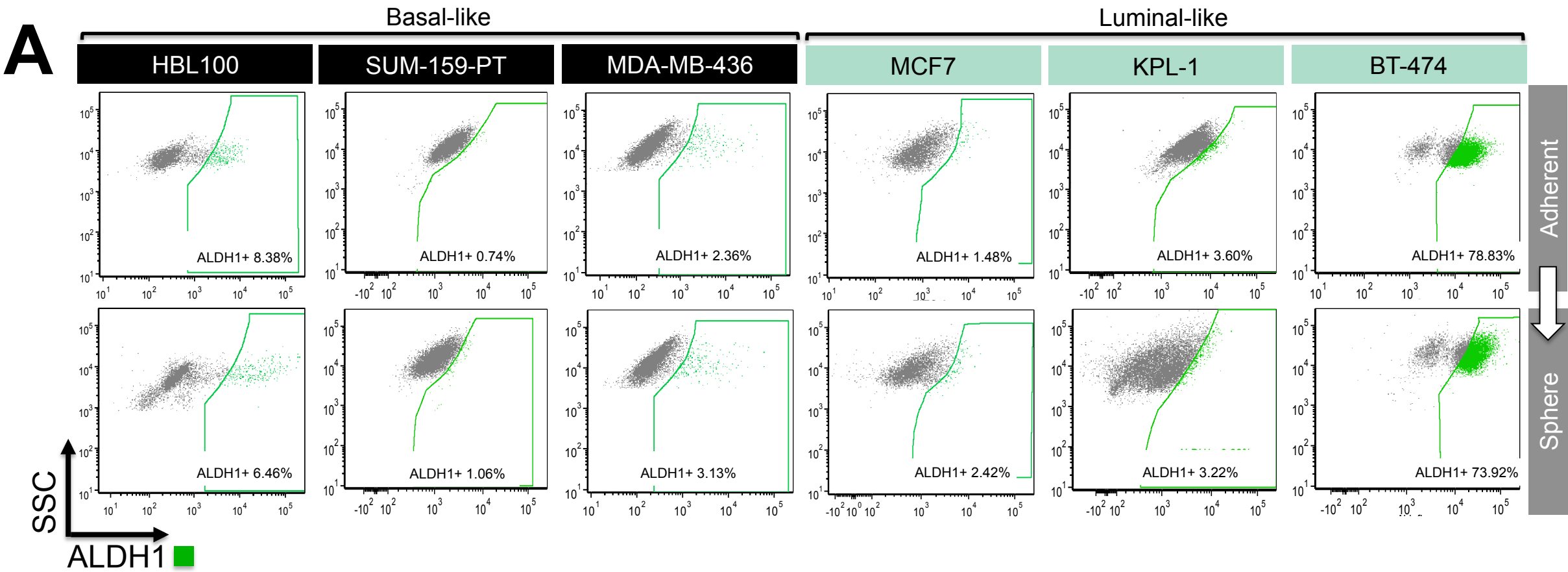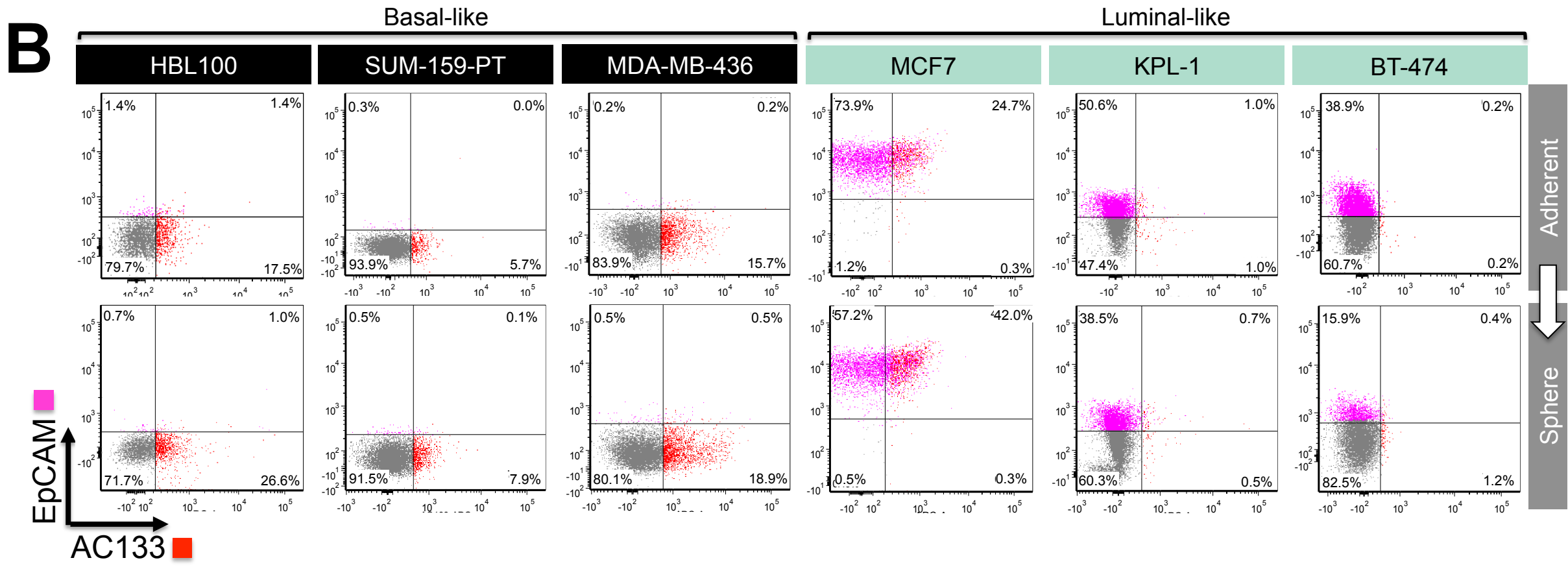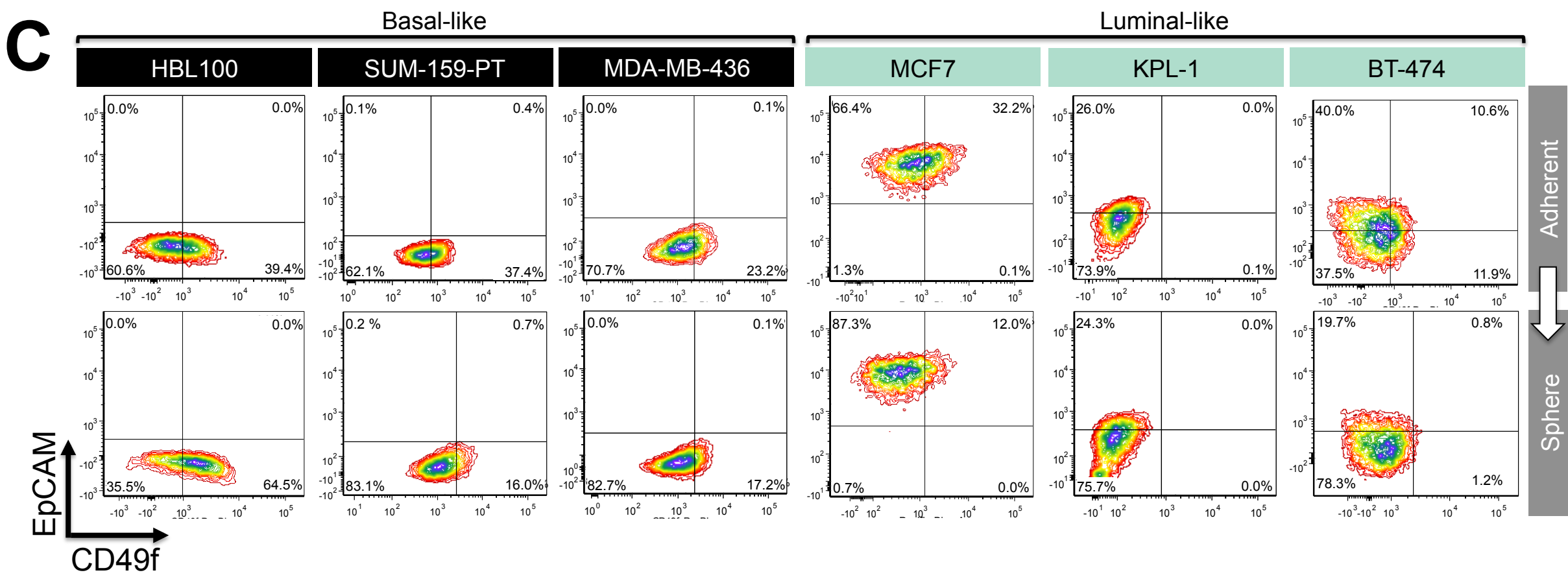

Supplement: Figure S3 — Changes in the activity of ALDH1 (A) and expression of EpCAM, CD133 (AC133) (B) and CD49f (C) with sphere culture. Adherent and sphere cultures were dissociated, stained with fluorescent antibody conjugates and analysed as described in Fig. 2. Representative data are depicted using dot or contour plots. Subpopulation frequencies shown represent the percentage of live cells. Quadrant gates were placed at the threshold of autofluorescence for respective adherent or sphere unstained control samples. (PDF) [file pone.0064388.s003.pdf]

Figure S4

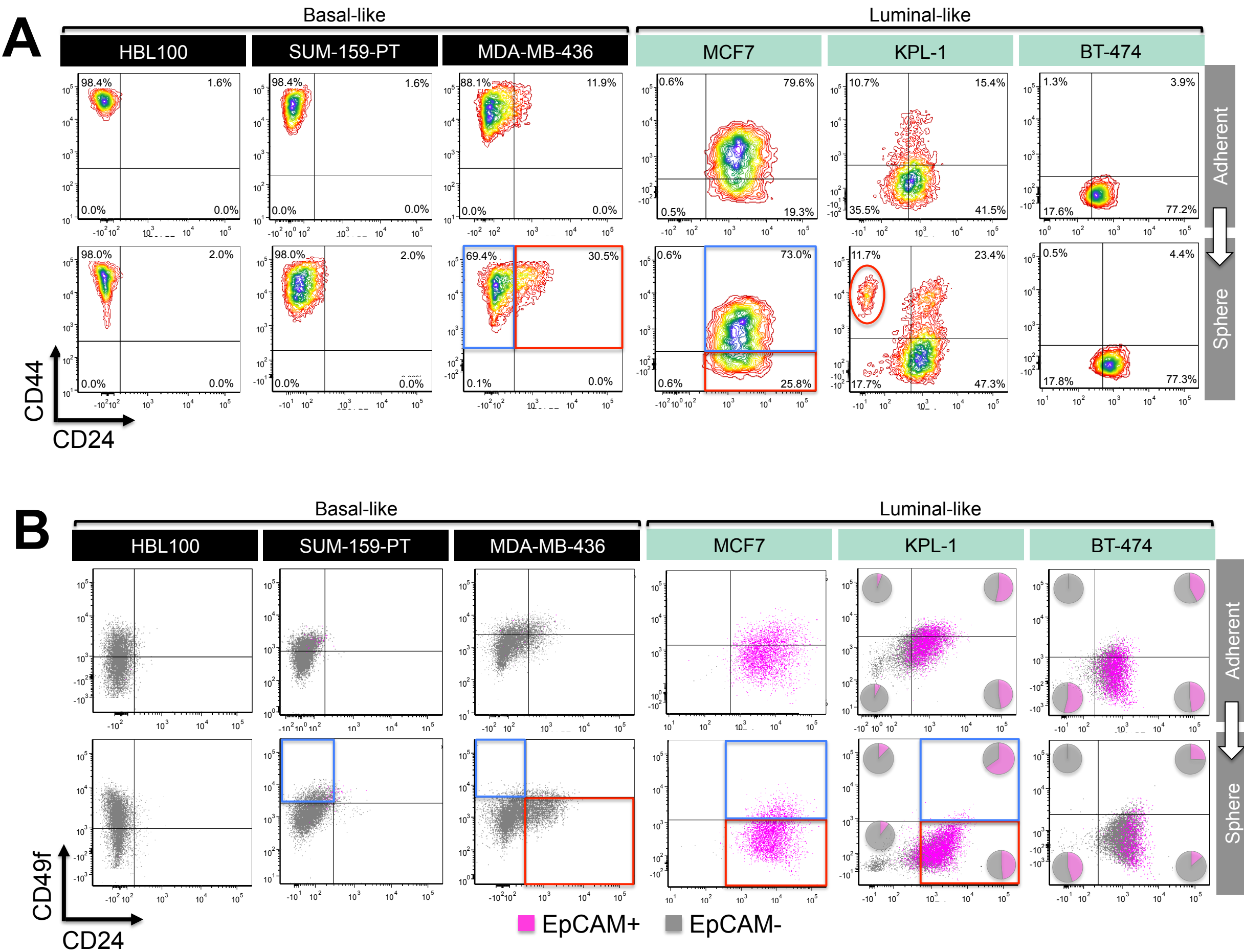

Supplement: Figure S4 — Changes in cell line differentiation states with sphere culture. Adherent and sphere cultures were dissociated, stained with fluorescent antibody conjugates and analysed as described in Fig. 2. (A) Changes in CD44/CD24 phenotypes with sphere culture. Representative data are depicted using contour plots. Subpopulation frequencies shown represent the percentage of live cells. Quadrant gates were placed at the threshold of autofluorescence for respective adherent or sphere unstained control samples. For KPL-1, the red circle indicates a consistent gain of a CD44+/CD24− subpopulation (not statistically significant by statistical analysis of quadrant gates but visually obvious). (B) Changes in CD49f/CD24/EpCAM distributions with sphere culture. Where the CD49f/CD24 distribution of EpCAM+ cells differed between adherent and sphere cultures, pie charts indicate the relative proportions of EpCAM+ and EpCAM- cells in each quadrant. 1×10^4^ events displayed on all plots. Red and blue quadrant colouring is transposed from Fig. 3 to indicate subpopulation frequencies that were consistent and statistically significant across biological replicates (red, increased in spheres compared to matched adherent cultures; blue, decreased in spheres). (PDF) [file pone.0064388.s004.pdf]

# Figure S5

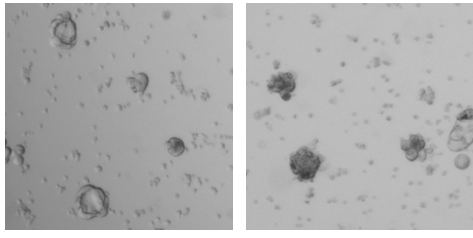

Supplement: Figure S5 — Light micrographs of hollow and solid spheres formed from fresh dissociations of normal human breast tissue. Images were taken at 100x magnification after 10 days in culture. (PDF) [file pone.0064388.s005.pdf]
