# Supplementary material for: In Vitro Analysis of Breast Cancer Cell Line Tumourspheres and Primary Human Breast Epithelia Mammospheres Demonstrates Inter- and Intrasphere Heterogeneity
Source: PLoS One. 2013 Jun 4;8(6):e64388. doi: 10.1371/journal.pone.0064388 (PMC3672101; doi:10.1371/journal.pone.0064388)
Supplement: Table S1 — Breast cancer cell lines: growth media and sphere-forming capacity. Cell lines were cultured as monolayers in the media indicated. Sphere-forming capacity (SFC) was then determined in triplicate on at least 2 occasions by seeding a standardised number of cells in sphere-promoting conditions (see materials and methods), then counting the number of spheres at 7 d relative to the number of parent cells seeded: ‘−’ = no spheres observed, ‘+’ = <0.01% and ‘++’ >0.01%. SFC was then correlated with adherent growth media, and published data (intrinsic molecular subtypes, tumourigenicity in mouse xenograft assays and 3D in vitro morphology in laminin-rich extracellular matrix (lrECM)), however we found no obvious association of any of these parameters with in vitro tumoursphere-forming capacity (SFC). (PDF) [file pone.0064388.s006.pdf]

Table S1

Cell Line	Growth Media	SFC	Subtype [28-31]	Tumourigenicity [28,32]	3D lrECM morphology [33]
MDA-MB-468	DMEM + FCS-10	-	BA	Yes	Grape-like
HCC1937	RPMI + FCS-10	-	BA		
PMC-42 ET*	RPMI + FCS-10	-	BA		
SVCT*	DMEM + FCS-10 + I-10 + HC-0.5	-	BA		
MCF10A	DMEM/F12 + HS-5 + EGF-0.02 + CTX-0.1 + I-10 + HC-0.5	-	BB	No	Round
BT-549	RPMI + FCS-10 + I-10	-	BB/C-low	Yes	Stellate
MDA-MB-157	DMEM + FCS-10	-	BB/C-low	Yes	Stellate
MDA-MB-231	DMEM + FCS-10	-	BB/C-low	Yes	Stellate
BT-483	RPMI + FCS-20+ I-10	-	Lum	Yes	Mass
MDA-MB-134-VI	DMEM + FCS-10	-	Lum	Yes	
MDA-MB-453	DMEM + FCS-10	-	Lum, ERBB2+	No	Grape-like
MDA-MB-330	DMEM + FCS-20+ EGF-0.03 + I-15	-	Lum, ERBB2+	No	
UACC-812	DMEM + FCS-10 + EGF-0.02	-	Lum, ERBB2+		Grape-like
BT-20	DMEM + FCS-10	+	BA	Yes	
Hs 578T	DMEM + FCS-10	+	BB/C-low	No	Stellate
HBL100	DMEM + FCS-10	++	BB/C-low	No/Weak	Stellate
MDA-MB-435	DMEM + FCS-10	++	BB/C-low	Yes	
SUM-159-PT	F12 + FCS-10+ I-10 + HC-0.5	++	BB/C-low	Yes	
MDA-MB-436	DMEM + FCS-10 + I-10	++	BB/C-low	Yes/Weak	Stellate
ZR751	RPMI + FCS-10	++	Lum	Yes	Grape-like
MCF7	DMEM + FCS-10+ I-10	++	Lum	Yes	Mass
T47D	RPMI + FCS-10+ I-20	++	Lum	Yes	Mass
KPL-1	DMEM + FCS-10	++	Lum	Yes	
MDA-MB-175-VII	DMEM + FCS-10	++	Lum	Yes	
BT-474	RPMI + FCS-10+ I-10	++	Lum, ERBB2+	Yes	Mass
SK-BR-3	McCoy's 5a + FCS-10	++	Lum, ERBB2+	Yes/Weak	Grape-like

Cell line intrinsic subtypes were designated based on published reports, considering gene expression profiling and cytokeratin phenotypes. The subtypes of PMC-42 ET and SVCT (*) were not previously published, and were classified in our laboratory using surrogate immunohistochemical markers, as previously described[29] (data not shown).

Abbreviations: BA = basal A; BB = basal B; C-low = claudin-low; Lum = Luminal; Lum, ERBB2+ = Luminal, HER2-amplified. RPMI, DMEM, DMEM/F12, F12, McCoy's 5a (base media; Gibco). lrECM = laminin-rich extracellular matrix; FCS-10/20 (10 or 20% foetal calf serum; Gibco); HS-5 (5% Horse serum; Gibco); I-10/20 (10 or 20 ug/mL insulin; Sigma-Aldrich); HC-0.5 (0.05 ug/mL hydrocortisone; Sigma); EGF-0.02/0.03 (0.02 or 0.03 ug/mL recombinant human epidermal growth factor; Sigma); CTX-0.1 (0.1ug/mL cholera toxin; Sigma-Aldrich). Note all media supplemented with 1x antibiotic/antimycotic (Gibco).
